# Study on the visible-light-induced photokilling effect of nitrogen-doped TiO_2 _nanoparticles on cancer cells

**DOI:** 10.1186/1556-276X-6-356

**Published:** 2011-04-21

**Authors:** Zheng Li, Lan Mi, Pei-Nan Wang, Ji-Yao Chen

**Affiliations:** 1 Key Laboratory of Micro and Nano Photonic Structures (Ministry of Education), Department of Optical Science and Engineering, Fudan University, Shanghai 200433, China; 2 Surface Physics Laboratory (National Key Laboratory), Department of Physics, Fudan University, Shanghai 200433, China

## Abstract

Nitrogen-doped TiO_2 _(N-TiO_2_) nanoparticles were prepared by calcining the anatase TiO_2 _nanoparticles under ammonia atmosphere. The N-TiO_2 _showed higher absorbance in the visible region than the pure TiO_2_. The cytotoxicity and visible-light-induced phototoxicity of the pure- and N-TiO_2 _were examined for three types of cancer cell lines. No significant cytotoxicity was detected. However, the visible-light-induced photokilling effects on cells were observed. The survival fraction of the cells decreased with the increased incubation concentration of the nanoparticles. The cancer cells incubated with N-TiO_2 _were killed more effectively than that with the pure TiO_2_. The reactive oxygen species was found to play an important role on the photokilling effect for cells. Furthermore, the intracellular distributions of N-TiO_2 _nanoparticles were examined by laser scanning confocal microscopy. The co-localization of N-TiO_2 _nanoparticles with nuclei or Golgi complexes was observed. The aberrant nuclear morphologies such as micronuclei were detected after the N-TiO_2_-treated cells were irradiated by the visible light.

## Introduction

Semiconductor titanium dioxide (TiO_2_) has been widely studied as a photocatalyst for its high chemical stability, excellent oxidation capability, good photocatalytic activity, and low toxicity [1-4]. Under the irradiation of ultraviolet (UV) light with the wavelength shorter than 387 nm (corresponding to 3.2 eV for the band gap of anatase TiO_2_), the electrons in the valence band of TiO_2 _can be excited to the conduction band, thus creating the pairs of photo-induced electron and hole. Then, the photo-induced electrons and holes can lead to the formation of various reactive oxygen species (ROS), which could kill bacteria, viruses, and cancer cells [5-10].

In recent years, TiO_2 _attracted more attention as a photosensitizer in the field of photodynamic therapy (PDT) due to its low toxicity and high photostability [2,3]. However, TiO_2 _can be activated by UV light only, which hinders its applications. Improvement of the optical absorption of TiO_2 _in the visible region by dye-adsorbed [11,12] or doping [13,14] methods will facilitate the practical application of TiO_2 _as a photosensitizer for PDT. When using dye-adsorbed method, the dyes such as hypocrellin B [11] and chlorine e6 [12] themselves are well-known PDT sensitizers and will have influence on the PDT efficiency of TiO_2_. For doping method, anionic species are preferred for the doping rather than cationic metals which have a thermal instability and an increase of the recombination centers of carriers [14]. In addition, cationic metals themselves always present cytotoxicity. Therefore, anionic species doping, especially nitrogen doping, is mostly adopted to improve the absorption of TiO_2 _in the visible region.

In the present work, the nitrogen-doped TiO_2 _(N-TiO_2_) nanoparticles were used as the photosensitizer to test its photokilling efficiency for three types of cancer cell lines. The N-TiO_2 _nanoparticles were prepared by calcining pure anatase TiO_2 _nanoparticles under ammonia atmosphere, which was an inexpensive method and easy to operate. The produced N-TiO_2 _nanoparticles have high stability and effective photocatalytic activity. Their absorption in the visible region was improved and their photokilling efficiency of cells under visible-light irradiation was compared with that of the pure TiO_2_. The intracellular distributions of these nanoparticles were measured by the laser scanning confocal microscopy (LSCM). The mechanisms of the photokilling effect were discussed.

## Methods

### Preparation and characterization of N-TiO_2 _nanoparticles

The anatase TiO_2 _nanoparticles (Sigma-Aldrich, St. Louis, MO, USA; particle size <25 nm) were calcined under ammonia atmosphere with various calcination parameters, such as temperature, gas flow rate, and calcination time, and then cooled down in nitrogen flow to the room temperature. Three N-TiO_2 _samples prepared with different calcination parameters were used in this work. Together with the pure TiO_2_, they are denoted as listed in Table 1. The crystalline phases of these samples were determined by Raman spectra (LABRAM-1B; HORIBA, Jobin Yvon, Kyoto, Japan). To evaluate their absorptions in the visible region, the ultraviolet-visible (UV/Vis) diffuse reflectance absorption spectra of these samples were measured with a Jasco V550 UV/Vis spectrophotometer (Jasco, Inc., Tokyo, Japan)

**Table 1:** Calcination parameters and the resulted crystalline phases of the TiO_2 _nanoparticles

Samples	Calcination parameters			Crystalline phases
	Temperature (°C)	Ammonia gas flow rate (L/min)	Time (min)	
Pure	-	-	-	Anatase
N-550-1	550	3.5	20	Anatase
N-550-2	550	7	10	Anatase
N-600-1	600	3.5	20	Rutile and anatase

Pure- and N-TiO_2 _nanoparticles were dispersed in Dulbecco's modified Eagle's medium with high glucose (DMEM-H), respectively, at various concentrations between 50 and 200 μg/mL. To avoid aggregation, these suspensions were ultrasonically processed for 15 min before using.

### Cell culture

The human cervical carcinoma cells (HeLa), human hepatocellular carcinoma cells (QGY), or human nasopharyngeal carcinoma cells (KB) procured from the Cell Bank of Shanghai Science Academy (Shanghai, China) were grown in 96-well plates or Petri dishes in DMEM-H solution supplemented with 10% fetal calf serum in a fully humidified incubator at 37°C with 5% CO_2 _for 24 h. Then, the culture medium was replaced by TiO_2_-containing medium and the cells were incubated for 2 h in the dark. After the TiO_2 _nanoparticles deposited and adhered to the cells, the medium was changed to the TiO_2_-free DMEM-H solution supplemented with 10% fetal calf serum for further study.

### Measurements of photokilling effect and cytotoxicity

To examine the photokilling effect, the cells were irradiated with the visible light from a 150-W Xe lamp (Shanghai Aojia Electronics Co. Ltd., Shanghai, China). Two pieces of quartz lens were used to obtain a concentrated parallel light beam. An IR cutoff filter was set in the light path to avoid the hyperthermia effect. A 400-nm longpass filter was used to cut off the UV light. The visible-light power density at the liquid surface in cell wells was 12 mW/cm^2 ^as measured by a power meter (PM10V1; Coherent, Santa Clara, CA, USA). After irradiation with this visible light for 4 h, cells were incubated in the dark for another 24 h until further analysis were conducted. The cytotoxicity examinations were carried out with the same procedure as the photokilling effect examinations but without the light irradiation, i.e., the TiO_2_-treated cells were incubated in the dark for 28 h.

The cell viability assays were conducted by a modified MTT method using WST-8 [2-(2-methoxy-4-nitrophenyl)-3-(4-nitrophenyl)-5-(2,4-disulfophenyl)-2*H *tetrazolium, monosodium salt] (Beyotime, Jiangsu, China). Each well containing 100 μL culture medium was added with 10 μL of the WST-8 reagent solution, and the cells were then incubated at 37°C with 5% CO_2 _for 2 h. Subsequently, the absorbance was measured at 450 nm using a microplate reader (Bio-Tek Synergy™ HT; Bio-Tek^® ^Instruments, Inc., Winooski, VT, USA). The untreated cells were used as the control groups. The surviving fraction represents the ratio of the viable TiO_2_-treated cells relative to that of the control groups. It should be noted that the TiO_2_-containing DMEM-H solution will affect the absorbance value at 450 nm. Therefore, when measuring the cell viability, the absorbance values were measured as a reference before the WST-8 dyes were added. Each experiment was performed in triplicate and repeated three times.

### Confocal laser scanning microscopy

The cells grown in Petri dishes were incubated with 50 μg/mL TiO_2 _in DMEM-H for 10 h before the LSCM observation (Olympus, FV-300, IX71; Olympus, Tokyo, Japan). Hoechst 33342 (Beyotime) and BODIPY FL C_5_-ceramide complexed to BSA (Molecular Probes; Invitrogen Corporation, Eugene, OR, USA) were used as the indicators for nucleus and Golgi complex, respectively. Hoechst 33342 (0.5 μg/mL) or Golgi complex marker (5 μM) was added into the growth medium for 15 to 30 min to stain the nuclei or Golgi complexes, respectively.

The reflection images of the intracellular TiO_2 _nanoparticles and the fluorescence images of nuclei (or Golgi complexes) were simultaneously obtained by the LSCM in two channels with no filter for the reflecting light and a 585 to 640-nm bandpass filter for the fluorescence. A 488-nm continuous-wave (CW) Ar^+ ^laser (Melles Griot, Carlsbad, CA, USA) or a 405-nm CW semiconductor laser (Coherent) was used as the excitation source. A 60 × water objective was used to focus the laser beam to a spot of about 1 μm in diameter. The differential interference contrast (DIC) micrographs to exhibit the cell morphology were acquired in a transmission channel simultaneously. The three-dimensional (3D) distributions of TiO_2 _nanoparticles and nuclei (or Golgi complexes) were obtained using the z-scan mode of the microscope.

## Results and discussion

### Raman spectra of TiO_2 _nanoparticles

**Figure 1:**
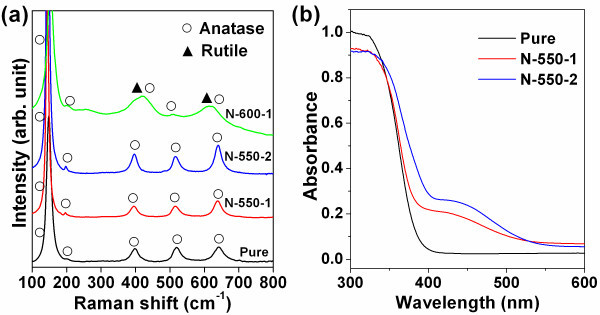
**Raman and UV/Vis diffuse reflectance spectra of the nanoparticle samples**. (**a**) Raman spectra of the pure and the three N-TiO_2 _nanoparticle samples. (**b**) Diffuse reflectance absorption spectra of samples pure, N-550-1, and N-550-2. Sample N-550-2 exhibited the highest absorbance in the visible region.

As shown in Table 1 and Figure 1a, the N-TiO_2 _samples N-550-1 and N-550-2 with the calcination temperature of 550°C, as well as the pure TiO_2_, exhibited a similar feature with five Raman peaks around 143, 197, 395, 514, and 640 cm^-1^, corresponding to the Raman fundamental modes of the anatase phase [15,16]. The Raman peaks for rutile phase [16] around 238, 420, and 614 cm^-1 ^appeared when the calcination temperature was 600°C as shown in the spectrum of the sample N-600-1. It can be concluded that the phase of the TiO_2 _nanoparticles would transform from anatase to rutile when the calcination temperature increased to 600°C. Such a phase transformation will result in a decrease of the photocatalytic ability for TiO_2 _powders [17,18]. Therefore, we only used samples N-550-1 and N-550-2 for further studies.

### Absorption spectra of TiO_2 _nanoparticles

Figure 1b shows the absorption spectra of the samples N-550-1 and N-550-2 and pure TiO_2_. Compared to the pure TiO_2_, the absorbances of N-550-1 and N-550-2 are higher in the visible region. However, the sample N-550-2 has the higher absorbance than N-550-1 in the region of 400 to 500 nm. Since N-550-1 and N-550-2 were calcinated at the same temperature and with the same amount of ammonia (flow rate times time), it seems that higher ammonia flow rate (N-550-2) could cause more absorption in the visible, which was expected to have higher photokilling efficiency of cells.

### Cytotoxicity and photokilling effect

**Figure 2:**
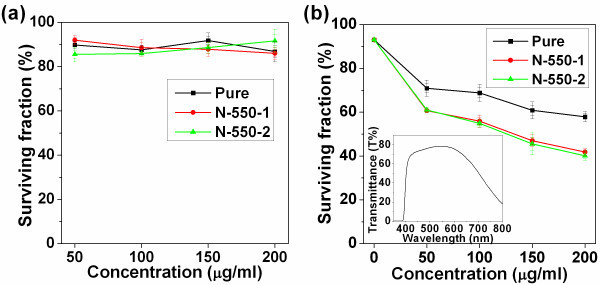
**Surviving fraction of treated and untreated HeLa cells**. (a) Surviving fraction of HeLa cells as a function of the concentration of TiO_2 _nanoparticles. HeLa cells were treated with 50, 100, 150, and 200 μg/mL TiO_2_, respectively, in the dark. The surviving fraction of untreated cells (control group) was set as 100%. (b) The photokilling effects of pure and N-TiO_2 _with different concentrations under visible irradiation. The inset is the transmittance spectrum of the combination of a 400 nm longpass filter and an IR cutoff filter used to acquire the visible-light irradiation from a Xe lamp.

**Figure 3:**
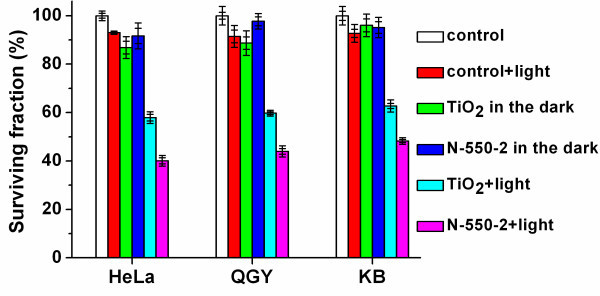
**The cytotoxicities and the photokilling effects of pure TiO**_**2 **_**and N-550-2 samples**. With the concentration of 200 μg/mL on HeLa, QGY, and KB cells. The control groups were also shown for comparison.

To evaluate the cytotoxicity of pure- and N-TiO_2 _nanoparticles, the TiO_2_-treated cells were further incubated in the dark for 28 h and the cell viability assays were then conducted. As shown in Figure 2a, all the surviving fractions of the treated HeLa cells were on the average values greater than 85% (with the concentration from 50 to 200 μg/mL). As shown in Figure 3, all the surviving fractions of the treated QGY or KB cells with the pure- or N-TiO_2 _concentration of 200 μg/mL in the dark were greater than 85%. These results indicated that the cytotoxicities of pure- and N-TiO_2 _nanoparticles were quite low. The cytotoxicities of these nanoparticles were quite similar, and there was no significant influence of the concentration on the cytotoxicity. Pure TiO_2 _is biocompatible with primary and cancer cells [4]. Nitrogen is an essential element of many biological molecules, such as proteins and nucleic acids. So, nitrogen is not toxic if it does not exceed the normal levels. It could be understood that a small amount of nitrogen doping would not lead to more cytotoxicity than pure TiO_2_.

The photokilling effects were measured as described in the experimental section. The surviving fractions of HeLa cells under visible-light irradiations for 4 h in dependence on the concentrations of pure- and N-TiO_2 _nanoparticles were shown in Figure 2b. As demonstrated in Figure 2b, the visible light showed very little photokilling effect on HeLa cells in the absence of any TiO_2 _(pure or N-doped) (at the 0 concentration). The surviving fractions (compared to the control cells without irradiation) were around 93%, which might be caused by the light irradiation, the fluctuant temperature during irradiation, and the experimental procedures. The spectrum of the light irradiated on cells (with filters) is also shown in the figure as an inset. It should be noted according to the spectrum in Figure 1b that the pure TiO_2 _nanoparticles still has some absorption around 400 nm though the band gap of TiO_2 _was reported to be 3.2 eV (corresponding to a wavelength of 387 nm). Therefore, pure TiO_2 _exhibited some photokilling effect under visible-light irradiation as shown in Figure 2b. However, the cells treated with N-TiO_2 _were killed more effectively than that with pure TiO_2_. The photokilling effects of samples N-550-1 and N-550-2 were quite similar although their absorption spectra showed some difference. It is also demonstrated in Figure 2b that the survival fractions decreased with the increasing concentrations of the TiO_2 _samples. It decreased to 40% for the cells treated with sample N-550-2 at a concentration of 200 μg/mL.

The photokilling effects of sample N-550-2 at a concentration of 200 μg/mL on QGY and KB cells were also measured as shown in Figure 3. Similar with the photokilling effect on HeLa cells, the QGY and KB cells treated with N-550-2 were also killed more effectively than that with pure TiO_2 _under the visible-light irradiation. The results revealed that the N-TiO_2 _might be applied to different cancers as a photosensitizer for PDT.

### ROS influence on the photokilling effect

**Figure 4:**
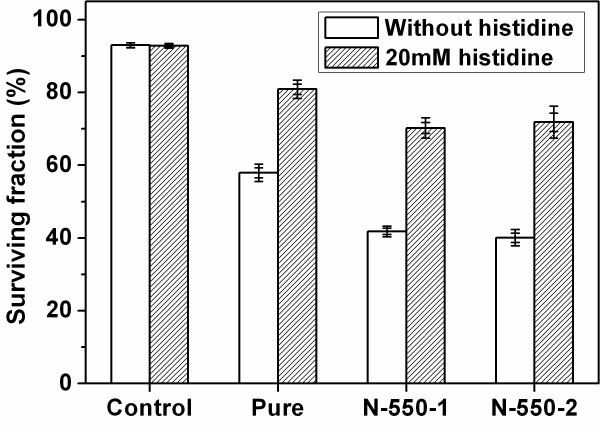
**Changes in the surviving fractions of the TiO**_**2**_**-treated HeLa cells with histidine**. The concentration of the three TiO_2 _samples is 200 μg/mL and L-histidine is 20 mM.

The mechanism of the photokilling effect for cancer cells caused by TiO_2 _nanoparticles is very complex. It has been identified that UV-photoexcited TiO_2 _in aqueous solution will result in formation of various ROS, such as hydroxyl radicals (**·**OH), hydrogen peroxide (H_2_O_2_), superoxide radicals (·O_2_^-^) and singlet oxygen (^1^O_2_) [19,20]. The ROS will attack the cancer cells and finally lead to the cell death. In order to study the function of ROS on the photokilling effect, the L-histidine, a quencher for both ^1^O_2 _and **·**OH [21-23], was added into the 96-well plates (20 mM) 30 min before the cells were irradiated by light. In the presence of 20 mM L-histidine, all the surviving fractions of the cells treated with pure- and N-TiO_2 _at a concentration of 200 μg/mL increased evidently as shown in Figure 4. These results are similar to the previous report for UV-photoexcited TiO_2 _[14]. It can be concluded that the ROS plays an important role on the photokilling effect, although we cannot tell which one played the main role. Further research is needed to figure out all the ROS influences.

### Distribution of TiO_2 _in cells

**Figure 5:**
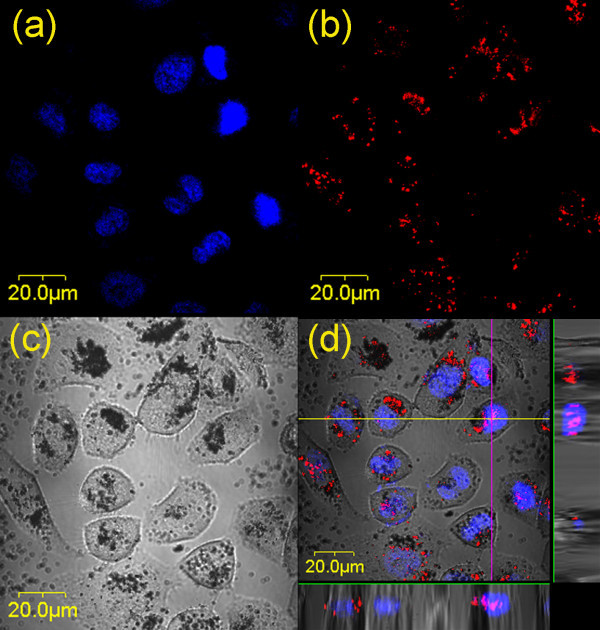
**Micrographs of the distributions of nuclei and TiO**_**2 **_**nanoparticles in HeLa cells**. **(a) **the distribution of nuclei (blue), **(b) **the distribution of TiO_2 _nanoparticles (red), **(c) **DIC micrograph, and **(d) **the merged image of (a), (b), and (c), in which the violet color denotes the co-localization of TiO_2 _nanoparticles with nuclei. The images displayed at the bottom and right side of (d) were the X-Z and Y-Z profiles measured along the lines marked in the main image, showing the 3D distributions of TiO_2 _nanoparticles and nuclei.

**Figure 6:**
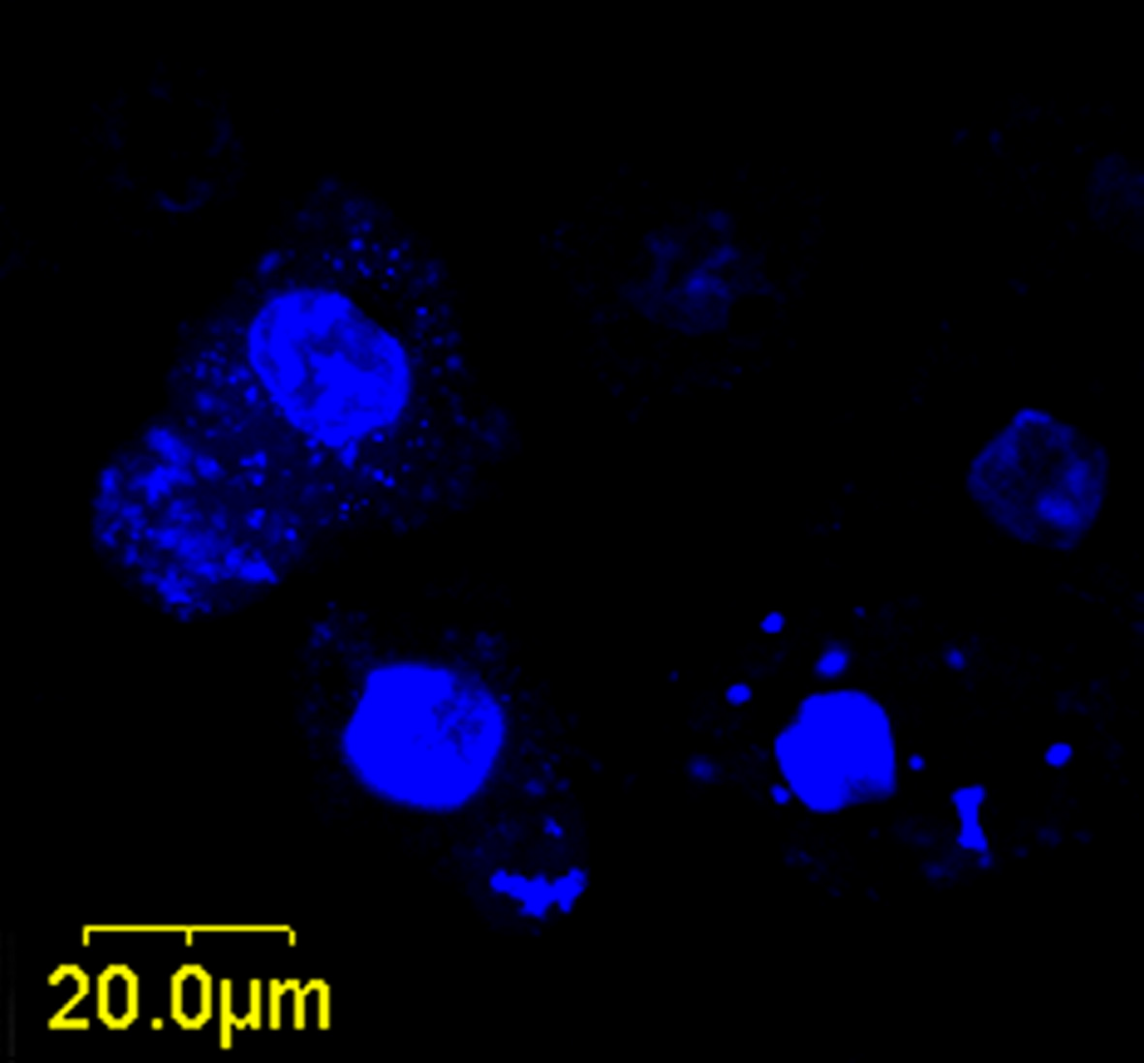
**Micrograph of the micronuclei of the HeLa cells**. Cultured with 50 μg/mL sample N-550-2 for 10 h and irradiated by a Xe lamp with a 400-nm longpass filter (12 mW/cm^2^) for 4 h. The micronuclei were observed.

As is well-known, light-excited TiO_2 _generates the electron-hole (e^-^/h^+^) pairs. The photogenerated carriers migrate to the particle surface and participate in various redox reactions there. Hence, the direct damage induced by photokilling effect would only occur at the sites of TiO_2 _particles. Therefore, it is of importance to know if the TiO_2 _nanoparticles were internalized into cells and how their intracellular distributions were. To find out the subcellular distribution of TiO_2 _nanoparticles, the TiO_2_-treated HeLa cells were stained with fluorescence indicators for Golgi complex and nucleus, respectively. Surprisingly, some TiO_2 _nanoparticles were found inside the nuclei as shown in Figure 5, where the HeLa cells were treated with (N-550-2, 50 μg/mL) and stained with nuclear indicator. When these N-TiO_2_-treated cells were irradiated by light from the Xe lamp with a 400-nm longpass filter (12 mW/cm^2^) for 4 h, some micronuclei were observed as shown in Figure 6. Since the TiO_2 _nanoparticles had entered into the nuclei of cells, the photoactivation effect could occur directly inside the nuclei, which might cause chromosomal damage or nucleus aberration. Micronuclei are usually formed from a chromosome or a fragment of a chromosome not incorporated into one of the daughter nuclei during cell division. This is an evidence of the direct damage to the nucleus resulted from the photoexcited N-TiO_2 _nanoparticles.

**Figure 7:**
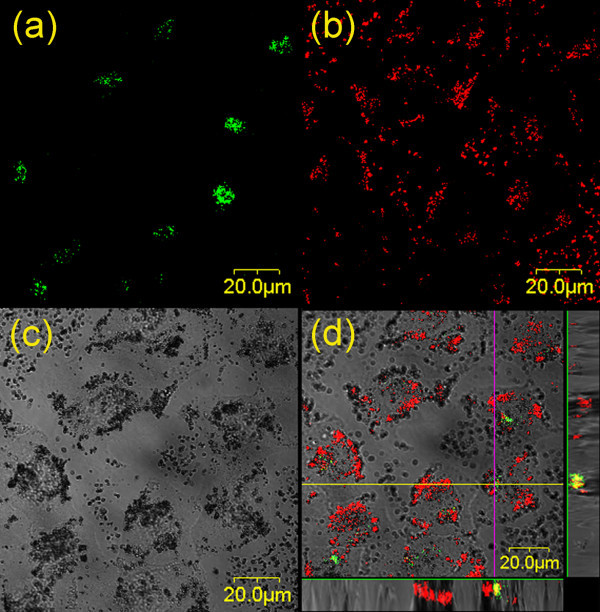
**Micrographs of the distributions of Golgi complexes and TiO**_**2 **_**nanoparticles in HeLa cells**. **(a) **The distribution of Golgi complexes (green), **(b) **the distribution of TiO_2 _nanoparticles (red), **(c) **differential interference contrast (DIC) micrograph, and **(d) **the merged image of (a), (b), and (c), in which the yellow color denotes the co-localization of TiO_2 _nanoparticles with Golgi bodies. The images displayed at the bottom and right side of (d) were the X-Z and Y-Z profiles measured along the lines marked in the main image, showing the 3D distributions of TiO_2 _and Golgi bodies.

Figure 7 is the confocal micrographs to show the distributions of Golgi complexes (fluorescence image) and TiO_2 _nanoparticles (reflection image) in HeLa cells. As shown in the merged image in Figure 7d, the TiO_2 _particles were not only found on the cell membrane but also in the cytoplasm. Some TiO_2 _nanoparticles aggregated around or in Golgi complexes. The co-localizations of TiO_2 _with Golgi complexes (yellow color) were observed. The cell viability might be influenced by the localization of TiO_2 _in Golgi complexes or other cell organelles, although there is no direct evidence found in this work.

## Conclusions

In the present work, N-TiO_2 _nanoparticles were prepared by calcination under ammonia atmosphere, which is an easily operative method and can achieve the product fruitfully. All the cytotoxicities of the pure- or N-TiO_2 _nanoparticles were quite low. The N-TiO_2 _samples showed higher absorbance and better photokilling effect than the pure TiO_2 _in the visible region. Therefore, the N-TiO_2 _has a higher potential as a photosensitizer for PDT of cancers_._

TiO_2 _is nonfluorescent and cannot be detected by fluorescence imaging. However, it can be monitored by the reflection imaging, which makes it convenient to record simultaneously with the fluorescence image using a LSCM. Co-localization of N-TiO_2 _nanoparticles with nuclei was observed. After visible-light irradiation, some micronuclei were detected as a sign of the nucleus aberration. Furthermore, ROS was found to play an important role on the photokilling effect for cells. However, the mechanisms for the photokilling effect on cancer cells should be investigated in details further.

## Competing interests

The authors declare that they have no competing interests.

## Authors' contributions

ZL carried out the experiments and drafted the manuscript. LM designed the project, participated in the confocal microscopy imaging, and wrote the manuscript. PW supervised the work and participated in the discussion of the results and in revising the manuscript. JC participated in the discussion of the results. All authors read and approved the final manuscript.

## References

[B1] Szacilowski K Macyk W Drzewiecka-Matuszek A Brindell M Stochel G Bioinorganic photochemistry: Frontiers and mechanisms Chem Rev 2005 105 2647 2694 10.1021/cr030707e 15941225

[B2] Warheit DB Hoke RA Finlay C Donner EM Reed KL Sayes CM Development of a base set of toxicity tests using ultrafine TiO_2 _particles as a component of nanoparticle risk management Toxicol Lett 2007 171 99 110 10.1016/j.toxlet.2007.04.008 17566673

[B3] Fabian E Landsiedel R Ma-Hock L Wiench K Wohlleben W van Ravenzwaay B Tissue distribution and toxicity of intravenously administered titanium dioxide nanoparticles in rats Arch Toxicol 2008 82 151 157 10.1007/s00204-007-0253-y 18000654

[B4] Carbone R Marangi I Zanardi A Giorgetti L Chierici E Berlanda G Podestà A Fiorentini F Bongiorno G Piseri P Pelicci PG Milani P Biocompatibility of cluster-assembled nanostructured TiO_2 _with primary and cancer cells Biomaterials 2006 27 3221 3229 10.1016/j.biomaterials.2006.01.056 16504283

[B5] Adams LK Lyon DY Alvarez PJ Comparative eco-toxicity of nanoscale TiO_2_, SiO_2_, and ZnO water suspensions Water Res 2006 40 3527 3532 10.1016/j.watres.2006.08.004 17011015

[B6] Thevenot P Cho J Wavhal D Timmons RB Tang LP Surface chemistry influences cancer killing effect of TiO_2 _nanoparticles Nanomed-Nanotechnol 2008 4 226 236 10.1016/j.nano.2008.04.001 PMC2597280 18502186

[B7] Brunet L Lyon DY Hotze EM Alvarez PJJ Wiesner MR Comparative photoactivity and antibacterial properties of C_60 _fullerenes and titanium dioxide nanoparticles Environ Sci Technol 2009 43 4355 4360 10.1021/es803093t 19603646

[B8] Choi O Hu ZQ Role of reactive oxygen species in determining nitrification inhibition by metallic/oxide nanoparticles J Environ Eng-Asce 2009 135 1365 1370 10.1061/(ASCE)EE.1943-7870.0000103

[B9] Lagopati N Kitsiou PV Kontos AI Venieratos P Kotsopoulou E Kontos AG Dionysiou DD Pispas S Tsilibary EC Falaras P Photo-induced treatment of breast epithelial cancer cells using nanostructured titanium dioxide solution J Photoch Photobio A 2010 214 215 223 10.1016/j.jphotochem.2010.06.031

[B10] Zhang DQ Li GS Yu JC Inorganic materials for photocatalytic water disinfection J Mater Chem 2010 20 4529 4536 10.1039/b925342d

[B11] Xu SJ Shen JQ Chen S Zhang MH Shen T Active oxygen species (^1^O_2_, O_2_^·-^) generation in the system of TiO_2 _colloid sensitized by hypocrellin B J Photoch Photobio B 2002 67 64 70 10.1016/S1011-1344(02)00263-4 12007469

[B12] Tokuoka Y Yamada M Kawashima N Miyasaka T Anticancer effect of dye-sensitized TiO_2 _nanocrystals by polychromatic visible light irradiation Chem Lett 2006 35 496 497 10.1246/cl.2006.496

[B13] Janczyk A Wolnicka-Głubisz A Urbanska K Stochel G Macyk W Photocytotoxicity of platinum(IV)-chloride surface modified TiO_2 _irradiated with visible light against murine macrophages J Photoch Photobio B 2008 92 54 58 10.1016/j.jphotobiol.2008.05.003 18555693

[B14] Janczyk A Wolnicka-Głubisz A Urbanska K Kisch H Stochel G Macyk W Photodynamic activity of platinum(IV) chloride surface-modified TiO_2 _irradiated with visible light Free Radical Bio Med 2008 44 1120 1130 10.1016/j.freeradbiomed.2007.12.019 18194674

[B15] Chen XB Lou YB Samia ACS Burda C Gole JL Formation of oxynitride as the photocatalytic enhancing site in nitrogen-doped titania nanocatalysts: Comparison to a commercial nanopowder Adv Funct Mater 2005 15 41 49 10.1002/adfm.200400184

[B16] Wang H Wu Y Xu BQ Preparation and characterization of nanosized anatase TiO_2 _cuboids for photocatalysis Appl Catal B 2005 59 139 146 10.1016/j.apcatb.2005.02.001

[B17] Mi L Xu P Wang PN Experimental study on the bandgap narrowings of TiO_2 _films calcined under N_2 _or NH_3 _atmosphere Appl Surf Sci 2008 255 2574 2580 10.1016/j.apsusc.2008.07.150

[B18] Wantala K Laokiat L Khemthong P Grisdanurak N Fukaya K Calcination temperature effect on solvothermal Fe-TiO_2 _and its performance under visible light irradiation J Taiwan Inst Chem E 2010 41 612 616 10.1016/j.jtice.2010.01.008

[B19] Daimon T Nosaka Y Formation and behavior of singlet molecular oxygen in TiO_2 _photocatalysis studied by detection of near-infrared phosphorescence J Phys Chem C 2007 111 4420 4424 10.1021/jp070028y

[B20] Tachikawa T Majima T Single-molecule detection of reactive oxygen species: application to photocatalytic reactions J Fluoresc 2007 17 727 738 10.1007/s10895-007-0181-5 17453327

[B21] Wade AM Tucker HN Antioxidant characteristics of L-histidine J Nutr Biochem 1998 9 308 315 10.1016/S0955-2863(98)00022-9

[B22] Schweitzer C Schmidt R Physical mechanisms of generation and deactivation of singlet oxygen Chem Rev 2003 103 1685 1757 10.1021/cr010371d 12744692

[B23] Redmond RW Kochevar IE Spatially resolved cellular responses to singlet oxygen Photochem Photobiol 2006 82 1178 1186 10.1562/2006-04-14-IR-874 16740059

